# Systematic Review of the Use of Phytochemicals for Management of Pain in Cancer Therapy

**DOI:** 10.1155/2015/506327

**Published:** 2015-10-20

**Authors:** Andrew M. Harrison, Fabrice Heritier, Bennett G. Childs, J. Michael Bostwick, Mikhail A. Dziadzko

**Affiliations:** ^1^Medical Scientist Training Program, Mayo Clinic, Rochester, MN 55905, USA; ^2^Department of Anesthesiology, CH du Forez, 42600 Montbrison, France; ^3^Mayo Graduate School, Mayo Clinic, Rochester, MN 55905, USA; ^4^Department of Psychiatry & Psychology, Mayo Clinic, Rochester, MN 55905, USA; ^5^Department of Anesthesiology, Mayo Clinic, Rochester, MN 55905, USA

## Abstract

Pain in cancer therapy is a common condition and there is a need for new options in therapeutic management. While phytochemicals have been proposed as one pain management solution, knowledge of their utility is limited. The objective of this study was to perform a systematic review of the biomedical literature for the use of phytochemicals for management of cancer therapy pain in human subjects. Of an initial database search of 1,603 abstracts, 32 full-text articles were eligible for further assessment. Only 7 of these articles met all inclusion criteria for this systematic review. The average relative risk of phytochemical versus control was 1.03 [95% CI 0.59 to 2.06]. In other words (although not statistically significant), patients treated with phytochemicals were slightly more likely than patients treated with control to obtain successful management of pain in cancer therapy. We identified a lack of quality research literature on this subject and thus were unable to demonstrate a clear therapeutic benefit for either general or specific use of phytochemicals in the management of cancer pain. This lack of data is especially apparent for psychotropic phytochemicals, such as the *Cannabis* plant (marijuana). Additional implications of our findings are also explored.

## 1. Introduction

Pooled prevalence of pain in cancer is greater than 50% [1]. This data is based on a systematic review of 52 research articles (out of a possible 4,737 articles) that spans 40 years of literature and includes patients after curative treatment, during cancer therapy, characterized as advanced/metastatic/terminal disease, and/or at all disease stages. One reason for the low article inclusion rate in this systematic review (1.1%) is the complex, multifactorial nature of cancer pain, which includes different mechanisms and targets [2]. In the context of this multifactorial nature and poor definition of cancer pain, stringent inclusion criteria were another reason for this low article inclusion rate. Although phytochemical therapy has historically been used as a treatment for cancer, treatment of cancer pain in general is challenging [3]. The use of phytochemical therapy for the treatment of cancer pain is further confounded by historical folklore and phytochemical isolates of poorly defined chemical composition. Specific plants and the phytochemicals from these plants have been investigated for their anti-inflammatory properties [4]. One example is the dried fruits of flowering shrub* Carissa carandas*. In this case, a specific plant containing potentially numerous compounds active against pain, as opposed to a specific phytochemical, was investigated. Numerous plants have also been used in traditional South African medicine for the treatment of pain [5]. However, rigorous scientific investigation into any specific phytochemicals these plants may contain for the specific treatment of pain is lacking. These examples are in contrast with examples of progress in chemotherapeutics.

The need for new chemotherapeutic agents with increased efficacy and decreased toxicity has led to the development of novel biologicals, such as antibody therapy, for the treatment of cancer [6]. This movement has resulted in some success, such as rituximab for the treatment of B cell-mediated lymphomas and leukemias. An extract of* Toxicodendron vernicifluum* (also known as* Rhus verniciflua* Stokes and the Chinese lacquer tree) has been shown to induce growth inhibition and apoptosis of hepatic tumor cells in cell culture [7]. Likewise, ursolic acid—found in the waxy peels of fruits, as well as some herbs and spices—has been demonstrated to induce apoptosis of melanoma cells in cell culture [8]. Thus, evidence exists for the use of phytochemicals as novel chemotherapeutic agents. The molecular signaling pathways and other mechanisms explaining these observations are slowly being elucidated [9, 10]. For example, a variety of natural inhibitors of the STAT3 signaling pathway, which results in the induction of apoptosis in both hematological and solid tumor cells, have been identified. Examples include betulinic acid, butein, caffeic acid, and capsaicin. However, the need for more agents with this level of success for the management of chemotherapeutic pain has resulted in other avenues of investigation [11], including phytochemicals and other natural products [12]. The rationale for this investigation includes epidemiological data from dietary intakes and* in vitro* experimentation.

While investigation into the use of phytochemicals for cancer chemotherapy is limited, exploration of phytochemicals for management of pain in cancer therapy is even more lacking. Specific extracts of the plant* Swertia corymbosa* have been isolated, identified, and shown to have dose-dependent therapeutic effects in mouse and rat models for the management of convulsions, sedation, and anxiety [13]. Numerous traditional herbs and phytochemicals have also already been investigated at the level of* in vivo* experiments and some clinical trials for neurodegenerative diseases, such as Alzheimer's disease [14]. In the context of these largely preclinical results, our objective was to perform a systematic review of the biomedical literature for the specific use of phytochemicals for the management of pain in cancer therapy in human subjects.

## 2. Methods

### 2.1. Background Definitions

One popular definition of phytochemicals (of the Kingdom Plantae) is “bioactive nonnutrient plant compounds in fruits, vegetables, grains, and other plant foods that have been linked to reducing the risk of major chronic diseases” [15]. For the purpose of this systematic review, as described in the search strategy below and the Discussion, fungochemicals (of the Kingdom Fungi) were also included. For the definition of management of pain in cancer therapy, both the direct and indirect antinociceptive effects of pain associated with antineoplastic treatment (oral mucositis, burns, neuropathy, enteritis, and proctitis)—as well as coanalgesic effects—were considered. The search strategy employed in this systematic review allowed for the inclusion of both whole plant products and specific plant extracts [16].

### 2.2. Data Sources and Searches

The internationally accepted Preferred Reporting Items for Systematic Reviews and Meta-Analyses (PRISMA) standard was used for this systematic review [17, 18]. A comprehensive search of 4 databases was conducted: PubMed (the National Library of Medicine), Ovid/MEDLINE (Wolters Kluwer), Scopus (Elsevier), and Web of Science (Thomson Reuters). English abstracts were searched from each database's inception through July 01, 2015. The search strategy was designed and conducted with a controlled vocabulary supplemented with keywords to search for studies of phytochemicals (“phytotherap∗”, “phytochemical∗”, “plant”, and “plants”), fungochemicals (mushr∗), pain (“pain” and “nociception”), and cancer (“cancer” and “neoplas∗”). Case reports, case series, case studies, controlled trials, and comparative studies were included in this search strategy. Meta-analysis, reviews, commentaries, and letters were excluded. All abstracts were screened by 1 reviewer (Mikhail A. Dziadzko) and potentially relevant articles in human subjects were identified for full-text review by 2 reviewers (Mikhail A. Dziadzko and Bennett G. Childs).

### 2.3. Study Selection

A study was eligible for inclusion if it examined the use of any phytochemical for the management of pain in cancer therapy in human subjects. Only interventional (nonobservational) studies with controls were included. Phytochemical derivatives were excluded, as were abstracts and articles not available in English.

### 2.4. Data Extraction and Quality Assessment

The primary outcome of this systematic review was response to phytochemicals in the management of pain in cancer therapy. The Cochrane Collaboration tool for assessing risk of bias was utilized to rank the quality of these papers [19]. Briefly, this tool includes scoring for (1) sequence generation, (2) allocation concealment, (3) blinding of participants and personnel, (4) blinding of outcome assessors, (5) incomplete outcome data, (6) selective outcome reporting, and (7) other sources of bias. The review of all full-text articles for inclusion based on this Cochrane Collaboration tool was performed by 2 reviewers (Mikhail A. Dziadzko and Fabrice Heritier).

### 2.5. Data Synthesis and Analysis

Data abstraction was coordinated and performed using the online systematic review software Covidence (Alfred Health, Monash University, Melbourne, Australia) [20]. For each study, relative risk was calculated by extracting the number of patients with dichotomous (binary) pain outcomes and comparing between the control and exposure groups. Statistical analysis, as well as forest plot and stacked bar chart generation, was performed using JMP (SAS, Cary, North Carolina). All confidence intervals (CIs) are reported at the 95% level. Final full-text article review was performed by 3 reviewers (Mikhail A. Dziadzko, Fabrice Heritier, and Bennett G. Childs).

## 3. Results

### 3.1. General Characteristics of Included Studies

**Figure 1:**
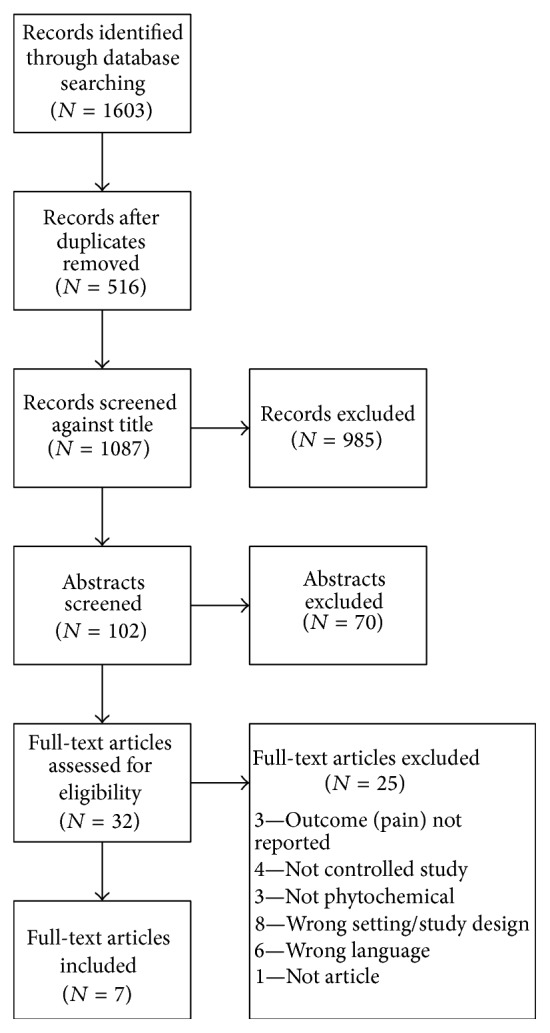
PRISMA flow chart.

A total of 1,603 abstracts were identified through initial database search (Figure 1). After removal of duplicate records (*N* = 516) and record screening against title (*N* = 985), a total of 102 abstracts remained for screening. Based on screening, 70 of these abstracts were excluded, leaving 32 full-text articles eligible for further assessment. A total of 25 of these full-text articles were excluded for wrong study design/setting (such as review or commentary) (*N* = 8), not being in the English language (*N* = 6), study not controlled (*N* = 4), no pain outcome reported (*N* = 3), not being a phytochemical (*N* = 3), or not being a research article (*N* = 1), leaving 7 full-text articles included in this systematic review.

### 3.2. Applied Methodology and Quality Assessment

**Figure 2:**
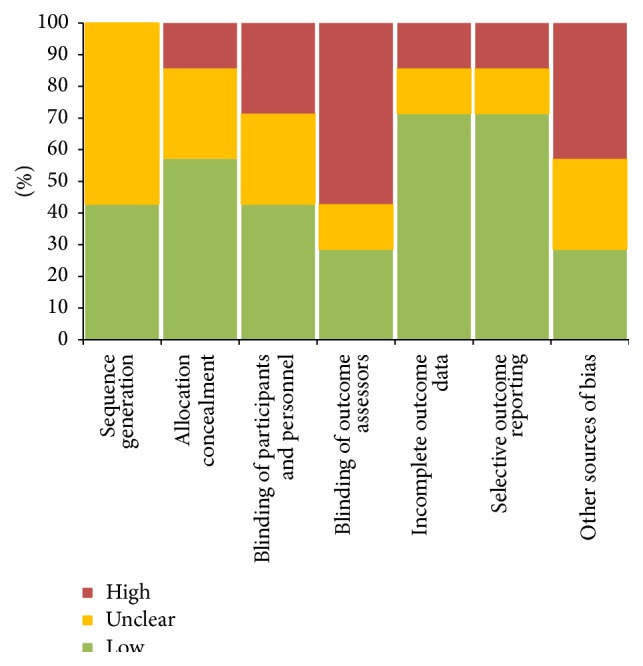
Stacked bar chart representation of results of the Cochrane Collaboration tool for assessing risk of bias across all studies.

The Cochrane Collaboration tool for assessing risk of bias was utilized to rank the quality of these 7 full-text articles to review author judgment (Table 1). For 6 of these 7 articles [21–26], at least 3 of the 7 Cochrane Collaboration tool scoring categories were ranked as “Low” risk of bias. In only 1 of these 7 articles were more than 2 of these scoring categories ranked as “High” risk of bias [27]. For all 7 of these full-text articles, at least 1 of these scoring categories was ranked as “Unclear” risk of bias. For better graphical representation across all studies, these results are also represented as a stacked bar chart (Figure 2).

### 3.3. Response to Phytochemicals for Management of Pain in Cancer Therapy

This systematic review synthesizes data for a different phytochemical in each of the 7 full-text articles examined (total *N* = 827) (Table 2). Briefly, 6 of the 7 studies used a placebo as the control. Study duration ranged from immediate effect to 12 months. Delivery methods included oral, ointment, oral solution, and subcutaneous injection. The only fungochemical examined was in the study by Costa Fortes and colleagues [24]. None of these research studies were performed in the United States and none of these 6 phytochemicals or 1 fungochemical is known to have a psychotropic effect.

**Figure 3:**
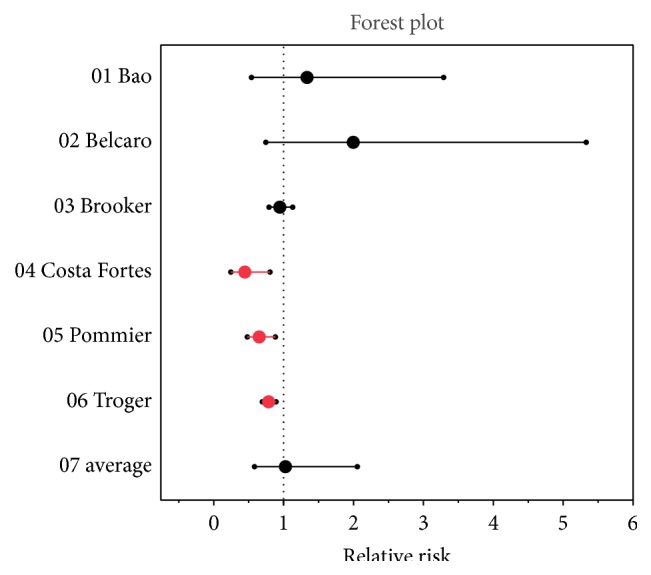
Forrest plot results of the systematic review. Note: Pawar and colleagues (the SAMITAL study) were excluded due to low *N* and methodological uncertainty.

The 1 study of SAMITAL by Pawar and colleagues [27] was excluded from relative risk analysis due to low *N* and methodological uncertainty, as this resulted in an inability to calculate an accurate relative risk (Table 3). The average relative risk of phytochemical compared to control for the included studies (total *N* = 800) was 1.03 [95% CI 0.59 to 2.06]. In other words (although not statistically significant), this relative risk indicates patients treated with phytochemicals were slightly more likely than patients treated with control to obtain successful management of pain in cancer therapy. To graphically assess response to phytochemicals in the management of pain in cancer therapy, a forest plot of relative risk for these 6 studies was generated (Figure 3).

## 4. Discussion

In this systematic review of the use of phytochemicals for management of pain in cancer therapy, we identified a lack of quality research literature on this subject (*N* = 7). While we were able to demonstrate a slight therapeutic benefit use of phytochemicals in the management of cancer pain, this benefit did not achieve statistical significance, which is a function of both the quality and marginal number of the studies that were acceptable for inclusion in a systematic review. The average relative risk of phytochemical compared to control for the included studies (total *N* = 800) was 1.03 [95% CI 0.59 to 2.06]. None of these research studies were performed in the United States and none of these 6 phytochemicals or 1 fungochemical is known to have a psychotropic effect.

Over 1,500 research articles were identified potentially examining the use of phytochemicals for management of pain in cancer therapy in initial database screening in this systematic review. However, only 32 research studies reached the level of full-text article assessment for eligibility. Ultimately, only 7 of these studies met final inclusion criteria. For example, although small (*N* = 27) [27], the study by Pawar and colleagues of SAMITAL, an oral solution of three botanical extracts (*Vaccinium myrtillus*,* Macleaya cordata*, and* Echinacea angustifolia*) for the relief of oral mucositis induced by chemotherapy and/or radiotherapy in oncological patients [28], has an elegant randomized, placebo-controlled, single-blind Phase II study design. However, due to low *N* and methodological uncertainty, this study could not be included in the relative risk analysis. Broadly, these results made formal efficacy score analysis for clinical practice recommendations with a standardized scoring system—such as the United States Preventive Services Task Force (USPSTF) grade (A, B, C, D, and I) and level of certainty (high, moderate, and low) or modified American Heart Association (AHA) class (I, IIa, IIb, and III) and level of evidence (A, B-R, B-NR, C, and E)—impossible [29, 30]. The lack of inclusion of any research study for any phytochemical from plants with potentially beneficial psychotropic effects, such as* Cannabis sativa* or* Cannabis indica* (marijuana), also raises concerns regarding the quality and comprehensiveness of current phytochemical literature and research related to the management of pain in cancer therapy.

In the case of the* Cannabis* plant, which contains the psychotropic chemical tetrahydrocannabinol (THC) and the weakly psychotropic chemical cannabinol (CBD), the potential benefits of this plant and these chemicals in management of pain, including for cancer therapy, have already been extensively explored [31]. One isomer of THC, dronabinol (trade name Marinol), has been approved by the United States (US) Food and Drug Administration (FDA) since 1985. Although beyond the immediate scope of this paper, a synthetic version of THC, nabilone (trade name Cesamet), has also been approved since 1985. In the US, another THC-rich* Cannabis* “extract,” nabiximols (trade name Sativex), and pure CBD isolate (trade name Epidiolex) are currently under review by the FDA for approval in the US [32]. For example, Sativex has been in Phase III trials since 2006. However, in the case of the* Cannabis* plant, its Schedule I drug status in the US (declared to have dangerous addictive potential and no redeeming medical value) since 1970 has made it difficult to study. Currently, much of the social and political controversy that surround the use of the* Cannabis* plant for pain is being fought out between the federal government and individual states, many of which have legalized the drug for medical use, recreational enjoyment, or both. For reference, several research articles on medical marijuana reach the level of the 32 full-text articles assessed for eligibility in the systematic review but were eventually excluded for one or more of the reasons described in the Results [33–36].

In the case of marijuana, some of the molecular signaling pathways and other mechanisms explaining the potential therapeutic benefit of marijuana in pain management in general have been partially elucidated through* in vitro*,* in vivo*, and human studies [37]. However, the lack of rigor of many of the research publications regarding the clinical efficacy of marijuana for the management of pain in cancer therapy is the result of social and political controversy surrounding its illicit drug status in the US and many other countries around the world [38]. In the case of the US state of Minnesota (where the main campus of Mayo Clinic is located), the production and distribution of medical marijuana were recently legalized at the state level which is currently in production in the form of oral whole plant extracts (containing THC, CBD, other cannabinoids, and other potentially psychotropic chemicals from the* Cannabis* plant) by at least one of the two state-approved manufacturers [39]. For reference, of the 1,500+ research abstracts examined in this systemic review, no other psychotropic phytochemical reached the level of more than 1 abstract and none were included for full-text review. Whether any of the phytochemicals examined in this systematic review will ultimately prove valuable for the management of pain in cancer therapy remains to be determined [40]. However, the additional sociological and political barriers to scientific investigation must be considered in the case of psychotropic phytochemicals, such as marijuana.

It is important to consider the potential application of phytochemicals from a holistic approach to medicine, as opposed to a disease-centric model for the treatment of medical illness. For example, the molecular signaling pathways and other mechanisms regulating the natural aging process, as well as chronic illnesses of aging, have been partially elucidated through an understanding of cellular senescence [41]. However, small molecules able to directly target senescent cells have yet to be identified. Beyond concerns regarding the quality and comprehensiveness of the current phytochemical literature and research regarding the management of pain in cancer therapy, as well as the specific case of psychotropic phytochemicals, there is also a need to consider the potential use of nonphytochemical fungochemicals in the management of pain in cancer therapy [42]. For example, the chaga mushroom (*Inonotus obliquus*) has longstanding historical value as a nonpsychotropic medicinal mushroom and is currently the subject of active research studies for its potential antioxidant, immuno-stimulating, anti-inflammatory, antinociceptive/pain, and anticancer properties [43–46]. Along these lines, the mechanisms of the 7 phytochemicals examined in this systematic review are thought to be primarily nociceptive, but the effect of these phytochemicals on the perception of pain is poorly understood.

*Limitations*. There are several limitations to this study. (1) As “filtered information” at the top of the evidence-based medicine pyramid, the conclusions of all systematic reviews are subject to the biases and confounders of the results of the research studies on which these conclusions are based [47]. (2) The word “phytochemical” or any of its permutations is used infrequently in the case of the* Cannabis* plant (marijuana). Thus, it is likely that this systematic review failed to identify relevant studies for consideration due to this controlled vocabulary [48]. (3) The lack of quality research studies regarding the specific use of phytochemicals for management of pain in cancer therapy limits the statistical power and conclusions that can be ascertained from any systemic review of this subject. For all of these reasons, more high-quality human research studies of the phytochemicals explored in this systematic review, as well as phytochemicals in general, are needed to determine the value of these individual phytochemicals and/or plant extracts in the management of pain in cancer therapy.

## 5. Conclusion

A lack of quality research literature on the subject of phytochemicals for management of pain in cancer therapy is identified in this systematic review. It is not currently possible to demonstrate a clear therapeutic benefit for either general or specific use of phytochemicals in the management of cancer pain. This lack of data is apparent for the psychotropic phytochemical-containing* Cannabis* plant (marijuana) but may only be a representative example of this problem due to the social and political controversy that surround this plant. There is also a need to consider the potential use of phytochemicals and nonphytochemical fungochemicals for applications ranging from holistic medicine to the natural aging process.

## Figures and Tables

**Table 1:** The Cochrane Collaboration tool for assessing risk of bias; review of author judgment of risk of bias for each item.

	Sequence generation	Allocation concealment	Blinding of participants and personnel	Blinding of outcome assessors	Incomplete outcome data	Selective outcome reporting	Other sources of bias
Bao et al., 2010 [21]	Low	Low	High	High	Low	Low	Unclear
Belcaro et al., 2014 [22]	Unclear	Unclear	Low	Low	Low	Unclear	High
Brooker et al., 2006 [23]	Unclear	Low	Low	Low	Low	Low	Low
Costa Fortes et al., 2010 [24]	Unclear	Unclear	Low	High	Low	Low	Low
Pawar et al., 2013 [27]	Unclear	High	Unclear	High	High	High	High
Pommier et al., 2004 [25]	Low	Low	Unclear	High	Low	Low	Unclear
Tröger et al., 2014 [26]	Low	Low	High	Unclear	Unclear	Low	Unclear

**Table 2:** Overview of the 7 full-text articles and associated phytochemicals included in this systematic review.

Study	*N*	Study design	Study duration	Phytochemical	Delivery	Patients	Country	Major study design bias
Bao et al. [21]	124	RCT, open label	Immediate intervention	Xiaozheng Zhitong Paste	Ointment	Multiple cancers with metastases	China	Control is not a placebo

Belcaro et al. [22]	80	RCT, open label	60 days	Meriva (lecithin delivery system of curcumin)	Oral	Chemo/radiotherapy postsurgical and multiple cancers	Italy	Heterogeneous cancer study population

Brooker et al. [23]	66	RCT, blinded	12 months	IH636 grape seed proanthocyanidin extract	Oral	Pain after high-dose radiotherapy for early breast cancer	UK	None

Costa Fortes et al. [24]	56	RCT, blinded	6 months	*Agaricus silvaticus* fungus extract	Oral	Postsurgical patients with colorectal cancer and pain	Brazil	Integrity of double-blinding unclear

Pawar et al. [27]	27	RCT, blinded	50 days	SAMITAL (three botanical extracts)	Oral solution	Pain from oral mucositis in patients treated for neck/head cancer	India	Control versus exposure group inequity

Pommier et al. [25]	254	RCT, open label	6 weeks	*Calendula* (plant)	Ointment	Pain after radiotherapy for breast carcinoma	France	Integrity of blinding questionable

Tröger et al. [26]	220	RCT, open label	12 months	Mistletoe	S/C injections	Pancreatic cancer	Serbia	Not blinded

Total	**827**							

**Table 3:** Relative risk results of the systematic review. Note: Pawar and colleagues (the SAMITAL study) were excluded due to low *N* and methodological uncertainty.

Study	*N*	Lower CI	Upper CI	Relative risk
Bao et al. [21]	124	0.54	3.29	1.34
Belcaro et al. [22]	80	0.75	5.33	2.00
Brooker et al. [23]	66	0.80	1.13	0.95
Costa Fortes et al. [24]	56	0.25	0.81	0.45
Pawar et al. [27]	(27)	N/A	N/A	N/A
Pommier et al. [25]	254	0.49	0.89	0.66
Tröger et al. [26]	220	0.70	0.90	0.79
Average	**800**	**0.59**	**2.06**	**1.03**
